# Body image and eating patterns among adolescents

**DOI:** 10.1186/1471-2458-13-1104

**Published:** 2013-12-01

**Authors:** Maria del Mar Bibiloni, Jordi Pich, Antoni Pons, Josep A Tur

**Affiliations:** 1 Research Group on Community Nutrition and Oxidative Stress, Universitat de les Illes Balears, Guillem Colom Bldg, Campus, E-07122 Palma de Mallorca, Spain; 2 CIBERobn (Fisiopatología de la Obesidad y la Nutrición) CB12/03/30038, E-07122 Palma de Mallorca, Spain

**Keywords:** BMI, FMI, Body image, Adolescents, Food intake, Eating patterns

## Abstract

**Background:**

Data on the association between body self-perception and eating patterns among adolescents are scarce. This study assessed the association between body image and eating patterns among normal-weight, overweight and obese adolescents.

**Methods:**

A cross-sectional survey (*n* = 1231; 12–17 years old) was carried out in the Balearic Islands, Spain. Anthropometry, body image, socio-economic determinants, and food consumption were studied.

**Results:**

Fifty-one percent of boys and sixty percent of girls that wished to be thinner had less than or equal to 3 eating occasions per day. Overfat girls that wish to be thinner skipped breakfast more frequently than normal-fat girls. Overfat boys and girls that wished a thinner body reported lower consumption of several food groups than normal-fat adolescents and overfat boys satisfied with their own body image (i.e. breakfast cereals, pasta and rice dishes, other oils and fats, high fat foods, soft drinks and chocolates in boys; and dairy products and chocolates in girls).A restriction of Western diet foods and energy intake was associated with a wish to be thinner among overfat adolescents.

**Conclusions:**

Many overfat boys were satisfied with their body image while practically all overfat girls reported wishing a thinner body. Meal patterns and food consumption were associated with body dissatisfaction and overfat status among adolescents.

## Background

Adolescence is a transitional stage and many changes take place at physiological and behavioural levels. Among adolescents, the prevalence of overweight and obesity has risen greatly worldwide [1,2], and among the Balearic Islands’ adolescents the prevalence of overweight (19.9% boys and 15.5% girls) and obesity (12.7% boys and 8.5% girls) should take into consideration [3]. Adolescent obesity is associated with significant immediate and long term health risks, and also predicts obesity in adulthood and increase risk of adult morbidity and mortality [1,2].

A pattern of healthy eating habits and adequate physical activity during adolescence reduces the risk of major chronic diseases [4-6]. However, a high intake of total fat, saturated fat and sodium, a low intake of vitamins and minerals, and a low consumption of fruits and vegetables are usual dietary patterns among adolescents [7-9], and only a small proportion of the Balearic Islands’ adolescents met the requirements of dietary fibre, folate, iodine, total fat, saturated fat, polyunsaturated fatty acid, total carbohydrate, and fruit and vegetables [10].

It has been pointed out that people with higher relative weight usually underreported their food intake [11]. However, controversial results have been reported on the association between food consumption and overweight and obesity, which can be attributed to overestimation of healthy foods and underestimation of unhealthy foods. Moreover, to avoid high-calorie foods has been associated with attempts to lose weight in adolescents [12,13].

Body image is a multidimensional construct central to emotional well-being in which the attitudinal component is satisfaction with body size, a factor associated with self-esteem [14]. During this period, the self-evaluation of body image and social patterns of beauty are factors that have a strong influence on eating habits [15-17]. Currently, there is a lack of data referring to the association between body self-perception and eating patterns among overweight and obesity in adolescents. Therefore these data are needed in order to design interventions to improve an effective nutrition and weight counselling among adolescents.

The aim of this study was to assess the association between body image and eating patterns among normal-weight, overweight and obese adolescents.

## Methods

### Study design

The study is a population-based cross-sectional nutritional survey carried out (2007–2008) in the Balearic Islands (Spain), a Mediterranean region.

### Selection of participants, recruitment and approval

A multicenter study was performed on Balearic Islands’ adolescents aged 12–17 years.The population was selected by means of a multiple-step, simple random sampling, first taking into account the location (Palma de Mallorca, Calvià, Inca, Manacor, Maó, Eivissa, Llucmajor, Santa Margalida, S’Arenal, Sant Jordi de Ses Salines) and then by random assignment of the schools within each city. Sample size was stratified by age and sex. The socio-economic variable was considered to be associated to geographical location and type of school. As the selection of schools was done by random selection and fulfilling quota, this variable was also considered to be randomly assigned.

To calculate a representative number of adolescents, the variable BMI with the greatest variance for this age group from the data published in the literature at the time the study was selected [18]. Sampling was determined for the distribution of this variable; and a confidence interval (CI) was established at 95% with an error ± 0.25. The total number of subjects (1500) was uniformly distributed in the cities and proportionally distributed by sex and age. Exclusion criteria used were: type 2 diabetes, pregnancy, alcohol or drug abuse, and non-directly related nutritional medical conditions.

The sample was oversized to prevent information loss and done when necessary to do the fieldwork in complete classrooms. In each school, classrooms were randomly selected among those of the same grade or level, and all the adolescents of one classroom were proposed to participate in the survey. A letter about the nature and purpose of the study informed parents or legal tutors and after receiving their written consent, the adolescents were considered for inclusion in the study. All responses to the questionnaires were filled in by adolescents. After finishing the field study, the adolescents who did not fulfil the inclusion criteria were excluded. Finally, the sample was adjusted by a weight factor in order to balance the sample in accordance to the distribution of the Balearic Islands’ population and to guarantee the representativeness of each of the groups, already defined by the previously mentioned factors (age and sex). The final number of subjects included in the study was 1231 adolescents (82% participation). Reasons for not participate were (a) the subject declined to be interviewed, and (b) the parents did not authorize the interview.

This study was conducted according to the guidelines laid down in the Declaration of Helsinki, and all procedures involving human subjects were approved by the Balearic Islands’ Ethics Committee (Palma de Mallorca, Spain).

### Anthropometry measurements

Height was determined using a mobile anthropometer (Kawe 44444, Asperg, Germany) measured to the nearest millimetre, with the subject’s head in the Frankfurt plane. Body weight was determined to the nearest 100 g using a digital scale (Tefal, sc9210, Rumilly, France), and subjects were weighed in bare feet and light underwear. Waist circumference (WC) and hip circumference (HC) were measured using a non-stretchable measuring tape (Kawe, 43972, France). The subjects were asked to stand erect in a relaxed position with both feet together on a flat surface. WC was measured as the smallest horizontal girth between the costal margins and the iliac crests at minimal respiration with measurements taken to the nearest 0.1 cm. HC was taken as the greatest circumference at the level of greater trochanters (the widest portion of the hip) on both sides with measurements taken to the nearest 0.1 cm. Triceps and subscapular skinfold thickness (ST) were measured on the right side of the using a Holtain skinfold caliper (Tanner/Whitehouse, Crosswell, Crymych, UK), and a mean of three measurements was used. Body fat percentage (%BF) was calculated from triceps and subscapular ST according to Slaughter et al. [19]. This equation has been proposed as the most accurate for estimation of %BF from ST in this particular population of adolescents [20]. Height and weight measures were used to calculate body mass index (BMI, kg/m^2^) and WC and height were used to calculate waist-to-height ratio (WHtR). %BF and height were used to calculate fat mass index (FMI; kg/m^2^).

### Defining overweight and obesity

Adolescents were age- and sex-specific classified using the BMI cut-offs developed and proposed by the International Obesity Task Force (IOTF) [21] and Cole et al. [22] definitions, and then subjects were classified as normal-fat and overfat according to their FMI using sex-specific cut-offs proposed for adolescents: 4.58 kg/m^2^ in boys and 7.76 kg/m^2^ in girls [23]. Thus, adolescents were classified into five weight and fat groups as following:

1) Underweight and normal-weight normal-fat (BMI for age and sex < P85; FMI < 4.58 kg/m^2^ in boys, FMI < 7.76 kg/m^2^ in girls).

2) Normal-weight overfat (BMI for age and sex < P85; FMI ≥ 4.58 kg/m^2^ in boys, FMI ≥ 7.76 kg/m^2^ in girls).

3) Overweight normal-fat (BMI for age and sex > P85 and < P97; FMI < 4.58 kg/m^2^ in boys, FMI < 7.76 kg/m^2^ in girls).

4) Overweight overfat (BMI for age and sex equivalent to > P85 and < P97; FMI ≥ 4.58 kg/m^2^ in boys, FMI ≥ 7.76 kg/m^2^ in girls).

5) Obesity (BMI for age and sex ≥ P97).

### Body image

Perceived body image was measured using the Stunkard scale [24], which consists of silhouette drawings ranging from 1 to 9 with monotonic increments in overweight percentage where 1 is the leanest and 9 is the heaviest. Separate figures for boys and girls were used. Participants were asked to identify of the 9 body figures: (a) ‘Which silhouette looks most like yourself?’ and (b) ‘Which silhouette would you like to look like?’ The difference between perceived body image and wished body image was used to determine the level of dissatisfaction with current body image. Values other than zero represent dissatisfaction with perceived body image. A positive value was indicative of the participant’s wish to be thinner than his/her perceived current size, while a negative value reflected the participant’s wish to be thicker than his/her current perceived size [25,26].

### Dietary assessment

Dietary assessment was assessed by using a validated [27] semi-quantitative food-frequency questionnaire (FFQ) covering 145 items (118 of the original validated FFQ plus the most characteristic Balearic Islands foods in order to make easy the interviewee answer). The FFQ evaluated average consumption over the past year. To prevent seasonal variations, the questionnaire was administered in the warm season (May-September) and in the cold season (November-March). Food consumption frequency was based on times that food items were consumed (per day, week or month). Consumption <1/month was considered no consumption. Daily food consumption (g/d) was determined by dividing the reported amount (g) of food consumed by the frequency of intake (d). Volumes and portion sizes were reported in natural units, household measures or with the aid of a manual of sets of photographs [28]. The 145 foods items from the FFQ were reduced to twenty-eight food groups, which may have practical importance in daily diet and clinical practice with Mediterranean youths [29,30].

Well-trained dieticians administered, verified and quantified all dietary questionnaires. To estimate volumes and portion sizes, the household measures found in the subjects’ own homes were used. Conversion of food into nutrients was done using a computer program (ALIMENTA®, NUCOX, Palma, Spain) based on Spanish [31,32] and European [33] food composition tables and complemented with food composition data available for Majorcan food items [34]. As an identification of misreporters: an energy intake (EI)/basal metabolic rate (BMR) ratio of <0.92 (boys) and <0.85 (girls) was considered to represent underreporters [35], and an EI:BMR ≥2.4 as overreporters [36].

### Assessment of meal patterns

The number of daily meals and snacks was calculated from the total eating occasions that participants declared among the following: breakfast, mid-morning snack, lunch, mid-afternoon snack, dinner, before going to sleep, others. Three groups of eating frequency were considered: ≤3, 4 and ≥5 times/d. Information about breakfast habit (yes; occasionally; no) was also collected.

### Assessment of socioeconomic factors

Socio-demographic factors were recorded using a questionnaire that included age group, parental education level (according to years and education type: low, <6 years; medium, 6–12 years; high, >12 years), and parental profession level (based on the occupation of parents and classified as low, medium and high, according to the Spanish Society of Epidemiology [37].

### Statistics

Analyses were performed with Statistical Package for the Social Sciences version 21.0 (SPSS, Inc., Chicago, IL, USA). Significant differences in energy intake were calculated by means of ANOVA, and significant differences in prevalence by means of χ^2^. We further applied multiple logistic regression analysis evaluating the association between body composition taking into account body image (normal-fat vs. overfat desiring to be thinner vs. overfat satisfied) with consumption frequencies of several food groups adjusted for potential confounders (age, parental educational level, parental socio-economic status, breakfast habit, number of daily meals and snacks). Level of significance for acceptance was *P* < 0.05.

## Results

### Body image according to body composition (BMI and FMI)

Table 1 shows the prevalence of normal-weight, overweight and obesity (BMI) according to overall adiposity (FMI) and desire to change weight. The three body weight groups obtained by the IOTF cut-offs (underweight and normal-weight, overweight, and obesity) were subgrouped according to presence or absence of overfat. Adolescents were classified into five groups as following: 73.2% underweight and normal-weight normal-fat, 2.1% normal-weight overfat, 6.7% overweight normal-fat, 11.9% overweight overfat and 6.1% obesity. The wish to change weight was assessed for each subgroup. Among boys, 39.1% of underweight and normal-weight normal-fat and 10.5% of overweight normal-fat adolescents reported to wish a thicker body shape; whereas 61.9% of normal-weight overfat, 82.4% of overweight overfat and 97.0% of obese boys reported to wish a thinner body shape. Among girls, around half of underweight and normal-weight normal-fat adolescents (47.8%) reported to wish a thinner body shape which increased according to the presence of excessive weight and/or excessive BF.

**Table 1:** Prevalence (%) of normal-weight, overweight and obesity associated with desiring for body weight change according to body weight (BMI) and adiposity (FMI) among Balearic Islands adolescents

**Categories**	**Boys (*n*** **= 574)**	**Girls (*n*** **= 657)**
**Underweight and Normal-weight**^**1**^		
**Normal-fat**^**4**^	**68.3**	**77.4**
Wants thinner body	14.3	47.8
Remain the same body	46.6	42.9
Wants thicker body	39.1	9.3
**Overfat**^**5**^	**4.0**	**0.5**
Wants thinner body	61.9	-
Remain the same body	38.1	-
Wants thicker body	0.0	-
**Overweight**^**2**^		
**Normal-fat**^**4**^	**3.6**	**9.3**
Wants thinner body	52.6	88.7
Remain the same body	36.8	11.3
Wants thicker body	10.5	0.0
**Overfat**^**5**^	**17.6**	**7.1**
Wants thinner body	82.4	93.6
Remain the same body	16.5	6.4
Wants thicker body	1.1	0.0
**Obesity**^**3**^	**6.5**	**5.8**
Wants thinner body	97.0	100.0
Remain the same body	3.0	0.0
Wants thicker body	0.0	0.0

*Abbreviations*: *BMI* body mass index; *FMI* fat mass index. ^1^Underweight and normal-weight (BMI-for age and sex <P85), ^2^overweight (BMI-for-age and sex >P85 and <P97) and ^3^obesity (BMI-for-age and sex ≥P97) as previously defined [21,22]. ^4^Normal-fat (boys: FMI <4.58 kg/m^2^; girls: FMI <7.76 kg/m^2^) and ^5^overfat (boys: FMI ≥4.58 kg/m^2^; girls: FMI ≥7.76 kg/m^2^) defined according to the previously proposed cut-offs [20]. Since the prevalence of overfat girls satisfied with own body shape was only 0.5% (n = 3) this group was not considered in this analysis.

### Meal patterns according to body composition and body image

Table 2 shows associations between meal patterns and body composition taking into account satisfaction with their body shape (normal-fat vs. overfat wishing to be thinner vs. overfat satisfied). It is important to note that most of overfat girls (96.6%) wished to be thinner, and an inverse association with number of daily meals and snacks and breakfast habit was found among them. Overfat boys that wished a thinner body shape (82.8%) also were more likely to have ≤3 eating occasions per day (50.8%) than their overfat satisfied and normal-fat counterparts.

**Table 2:** Meal patterns by body composition taking into account body image among Balearic Islands adolescents

	**Boys**				**Girls**		
	**Normal-fat**	**Overfat dissatisfied**^**1**^	**Overfat satisfied**^**2**^	***P***	**Normal-fat**	**Overfat dissatisfied**^**1**^	***P***
	**(*n*** **= 412)**	**(*n =*** **134)**	**(*n =*** **28)**		**(*n =*** **567)**	**(*n =*** **90)**	
**Breakfast habit**							
Yes	79.1	69.7	79.2	0.164	64.6	43.4	0.001
Occasionally	13.8	16.0	12.5		19.5	30.1	
No	7.2	14.3	8.3		15.9	26.5	
**Number of daily meals and snacks**							
≤3	18.9	50.8	21.7	<0.001	41.7	60.2	0.006
4	35.6	37.3	43.5		33.3	20.5	
≥5	45.6	11.9	34.8		25.3	19.3	

Values are %. Significant differences between groups were evaluated by χ^2^. ^1^Overfat desiring to be thinner and ^2^overfat satisfied with own body shape. Since the prevalence of overfat girls satisfied with own body shape was only 0.5% (n = 3) this group was not considered in this analysis.

### Food consumption according to body composition and body image

Associations between the food consumption and individual items and satisfaction with own body shapes were also evaluated (Table 3). Overfat boys that wished to be thinner were less likely to consume breakfast cereals, pasta and rice dishes, other oils and fats, high fat foods, soft drinks and chocolates than their satisfied and normal-fat counterparts. Compared with normal-fat girls, those who were overfat also reported to consume dairy desserts and chocolates with less frequency.

**Table 3:** Food consumption by body composition taking into account body image among Balearic Islands adolescents

		**Boys**				**Girls**		
**Food groups**	**Frequency categories**^**3**^	**Normal-fat**	**Overfat dissatisfied**^**1**^	**Overfat satisfied**^**2**^	***P***	**Normal-fat**	**Overfat dissatisfied**^**1**^	***P***
		**(*n*** **= 412)**	**(*n =*** **134)**	**(*n =*** **28)**		**(*n =*** **567)**	**(*n =*** **90)**	
**Dairy products**								
Milk	≥7 t/w	79.9	76.5	76.5	0.788	66.4	55.3	0.128
Yogurt and cheese	≥7 t/w	67.1	64.7	70.6	0.879	62.4	59.6	0.700
Dairy desserts	≥2 t/w	77.2	77.9	82.4	0.880	65.2	46.8	0.013
**Meat**								
Red meat	≥2 t/w	56.3	41.8	64.7	0.067	44.8	34.8	0.193
Poultry and rabbit	≥2 t/w	15.2	14.7	11.8	0.925	14.8	13.0	0.751
Sausages	≥5 t/w	52.4	52.2	58.8	0.874	47.6	45.7	0.804
	2-4 t/w	27.4	28.4	29.4	0.975	25.2	19.6	0.401
	≤4 t/m	20.1	19.4	11.8	0.699	27.3	34.8	0.279
**Fish and seafood**	≥2 t/w	17.4	25.4	17.6	0.317	15.7	6.5	0.096
**Eggs**	≥2 t/w	36.5	34.3	29.4	0.811	22.4	23.9	0.816
**Legumes**	≥2 t/w	19.7	22.1	5.9	0.317	17.5	10.6	0.234
**Cereals, grains and products**								
Bread	≥7 t/w	84.8	88.2	88.2	0.727	82.1	83.0	0.877
Breakfast cereals	≥5 t/w	53.3	35.3	47.1	0.028	30.1	31.9	0.799
Biscuits	≥5 t/w	22.8	17.6	17.6	0.594	23.0	14.9	0.205
Pasta and rice dishes	≥5 t/w	22.8	11.8	35.3	0.048	15.6	25.5	0.083
Pizza	≥2 t/w	18.7	10.3	11.8	0.213	11.0	14.9	0.429
**Fruits**								
Fresh fruits	≥2/day	30.1	32.4	29.4	0.932	30.8	31.9	0.876
Fruit juices	≥7 t/w	54.0	44.1	52.9	0.341	50.6	51.1	0.949
Canned fruits	≥2 t/w	9.3	10.3	5.9	0.855	4.8	6.4	0.641
**Vegetables**	≥2/day	7.3	7.4	5.9	0.976	12.9	14.9	0.696
**Nuts**	≥2 t/w	39.4	32.4	41.2	0.538	24.8	23.4	0.830
**Potatoes and tubercles**	≥2 t/w	42.9	47.1	41.2	0.807	33.3	29.8	0.623
**Fats**								
Olive oil	≥7 t/w	51.7	41.8	47.1	0.333	54.0	52.2	0.809
Other oils and fats	≥2 t/w	41.0	23.9	35.3	0.033	36.7	30.4	0.399
High fat foods	≥5 t/w	49.7	31.3	47.1	0.026	38.1	26.1	0.108
	2-4 t/w	24.3	32.8	41.2	0.138	28.4	32.6	0.550
	≤4 t/m	26.0	35.8	11.8	0.093	33.5	41.3	0.288
**Drinks**								
Soft drinks	≥5 t/w	59.4	40.3	52.9	0.018	41.8	43.5	0.827
	2-4 t/w	10.8	11.9	17.6	0.672	11.1	6.5	0.340
	≤4 t/m	29.9	47.8	29.4	0.019	47.1	50.0	0.709
Tea and coffee	≥2 t/w	10.8	13.4	23.5	0.258	16.7	13.0	0.527
Alcoholic beverages	≥1 t/w	8.9	5.9	11.8	0.637	3.7	4.3	0.845
**Sweets**								
Sweets	≥5 t/w	73.6	73.1	64.7	0.723	79.2	67.4	0.067
	2-4 t/w	14.6	11.9	17.6	0.787	13.0	19.6	0.214
	≤4 t/m	11.8	14.9	17.6	0.642	7.9	13.0	0.228
Chocolates	≥5 t/w	21.9	9.0	41.2	0.006	19.9	8.7	0.065
	2-4 t/w	11.5	13.4	5.9	0.681	11.8	6.5	0.284
	≤4 t/m	66.7	77.6	52.9	0.088	68.4	84.8	0.021

*Abbreviations*: *t/w* times/week, *t/m* times/month. Values are %. Significant differences between groups were evaluated by χ^2^. ^1^Overfat desiring to be thinner and ^2^overfat satisfied with own body shape. ^3^According to previously defined food consumption cut-offs [29,30]. Since the prevalence of overfat girls satisfied with own body shape was only 0.5% (n=3) this group was not considered in this analysis.

When energy intake (EI) was calculated, overfat adolescents (37%) misreported their EI more often than normal-fat peers (10%). Overfat adolescents who wished to be thinner showed a significant (*P* < 0.001) lower EI than overfat adolescents satisfied with their body shape and normal-fat adolescents, and overfat adolescents wishing to be thinner also showed significant (*P* < 0.001) lower energy intake from saturated fat acids than normal-fat peers (Table 4).

**Table 4:** Energy intake (mean values ± SD) by body composition taking into account body image among Balearic Islands’ adolescents

	**Boys**				**Girls**		
	**Normal-fat**	**Overfat dissatisfied**^**1**^	**Overfat satisfied**^**2**^	***P***	**Normal-fat**	**Overfat dissatisfied**^**1**^	***P***
	**(*n*** **= 412)**	**(*n =*** **134)**	**(*n =*** **28)**		**(*n =*** **567)**	**(*n =*** **90)**	
**Energy intake (kcal/d)**^**3**^	2378 ± 793	1849 ± 603	2361 ± 894	<0.001	1850 ± 606	1578 ± 607	<0.001
**Energy from AGS (%)**^**4**^	13.7 ± 3.8	12.6 ± 3.6	14.0 ± 4.9	<0.001	13.5 ± 4.1	12.7 ± 3.9	<0.001

^1^Overfat desiring to be thinner and ^2^overfat satisfied with own body shape. Since the prevalence of overfat girls satisfied with own body shape was only 0.5% (n=3) this group was not considered in this analysis. ^3^Significant differences between groups were evaluated by ANOVA. ^4^Significant differences between groups were evaluated by χ^2^.

Multiple logistic regression analysis (after adjustment by age, parental educational level, parental socio-economic status, breakfast habit, number of daily meals and snacks) showed that overfat that wished to be thinner were less likely to frequently eat red meat, pasta and rice dishes and other oils and fats than their satisfied and normal-fat counterparts (Table 5).

**Table 5:** Association between body image and food consumption among adolescents

		**Overfat desiring thinner body vs. overfat satisfied and normal-fat**^**2**^	
**Food groups**^**1**^	**Frequency categories**	**Boys**	**Girls**
**Dairy products**			
Milk	≥7 t/w vs. <7 t/w (ref.)	0.49 (0.19-1.29)	0.98 (0.41-2.34)
Yogurt and cheese	≥7 t/w vs. <7 t/w (ref.)	1.39 (0.62-3.12)	1.04 (0.43-2.50)
Dairy desserts	≥2 t/w vs. 2 t/w (ref.)	2.39 (0.92-6.18)	0.30 (0.13-0.70)**
**Meat**			
Red meat	≥2 t/w vs. 2 t/w (ref.)	0.35 (0.16-0.79)*	0.64 (0.27-1.53)
Poultry and rabbit	≥2 t/w vs. 2 t/w (ref.)	1.93 (0.58-6.47)	0.80 (0.23-2.75)
Sausages	≥5 t/w vs. ≤4 t/m (ref.)	1.61 (0.60-4.32)	1.12 (0.46-2.74)
	2-4 t/w vs. ≤4 t/m (ref.)	1.57 (0.57-4.35)	0.67 (0.23-1.98)
	≤4 t/m (ref.)		
**Fish and seafood**	≥2 t/w vs. 2 t/w (ref.)	2.24 (0.84-5.99)	0.29 (0.07-1.22)
**Eggs**	≥2 t/w vs. 2 t/w (ref.)	0.79 (0.34-1.88)	1.29 (0.51-3.28)
**Legumes**	≥2 t/w vs. 2 t/w (ref.)	1.27 (0.45-3.57)	0.49 (0.13-1.76)
**Cereals, grains and products**			
Bread	≥7 t/w vs. <7 t/w (ref.)	2.99 (0.90-9.98)	1.02 (0.34-3.01)
Breakfast cereals	≥5 t/w vs. <5 t/w (ref.)	0.56 (0.26-1.19)	1.42 (0.59-3.44)
Biscuits	≥5 t/w vs. <5 t/w (ref.)	0.90 (0.43-1.90)	1.09 (0.48-2.45)
Pasta and rice dishes	≥5 t/w vs. <5 t/w (ref.)	0.24 (0.08-0.76)*	2.45 (0.88-6.83)
Pizza	≥2 t/w vs. 2 t/w (ref.)	0.62 (0.21-1.83)	1.19 (0.31-4.59)
**Fruits**			
Fresh fruits	≥2/day vs. 2/day (ref.)	1.74 (0.78-3.88)	1.90 (0.79-4.59)
Fruit juices	≥7 t/w vs. <7 t/w (ref.)	0.62 (0.21-1.83)	1.65 (0.70-3.89)
Canned fruits	≥2 t/w vs. <2 t/w (ref.)	1.64 (0.43-6.19)	0.76 (0.12-4.71)
**Vegetables**	≥2/day vs. <2/day (ref.)	1.78 (0.36-8.83)	1.23 (0.40-3.81)
**Nuts**	≥2 t/w vs. 2 t/w (ref.)	0.50 (0.22-1.10)	1.31 (0.50-3.40)
**Potatoes and tubercles**	≥2 t/w vs. 2 t/w (ref.)	1.74 (0.79-3.88)	1.36 (0.57-3.26)
**Fats**			
Olive oil	≥7 t/w vs. <7 t/w (ref.)	0.87 (0.40-1.88)	0.97 (0.42-2.25)
Others oils and fats	≥2 t/w vs. <2 t/w (ref.)	0.41 (0.18-0.96)*	0.75 (0.32-1.80)
High fat foods	≥5 t/w vs. ≤4 t/m (ref.)	0.72 (0.28-1.86)	0.49 (0.15-1.64)
	2-4 t/w vs. ≤4 t/m (ref.)	1.50 (0.61-3.74)	0.76 (0.28-2.04)
	≤4 t/m (ref.)		
**Drinks**			
Soft drinks	≥5 t/w vs. ≤4 t/m (ref.)	0.43 (0.18-1.02)	1.46 (0.62-3.46)
	2-4 t/w vs. ≤4 t/m (ref.)	0.52 (0.15-1.77)	0.33 (0.04-2.87)
	≤4 t/m (ref.)		
Tea and coffee	≥2 t/w vs. 2 t/w (ref.)	2.14 (0.65-7.11)	0.74 (0.24-2.26)
Alcoholic beverages	≥1 t/w vs. <1 t/w (ref.)	0.79 (0.19-3.20)	3.32 (0.44-25.29)
**Sweet**			
Sweets	≥5 t/w vs. ≤4 t/m (ref.)	2.64 (0.71-9.77)	1.01 (0.30-3.42)
	2-4 t/w vs. ≤4 t/m (ref.)	1.36 (0.30-6.06)	1.38 (0.37-5.17)
	≤4 t/m (ref.)		
Chocolates	≥5 t/w vs. ≤4 t/m (ref.)	0.50 (0.13-1.90)	0.09 (0.01-0.74)*
	2-4 t/w vs. ≤4 t/m (ref.)	1.25 (0.41-3.80)	0.56(0.13-2.55)
	≤4 t/m (ref.)		

*Abbreviations*: *CI* confidence interval, *ref.* reference, *t/w* times/week, *t/m* times/month. ^1^According to previously defined food consumption cut-offs [29,30]. ^2^Multivariate analysis (multiple logistic regression analysis considering the simultaneous effect of all explanatory variables) adjusted by age, parental educational level, parental socio-economic status, breakfast habit and number of daily meals and snacks (significant differences: *P < 0.05; **P < 0.01). Since the prevalence of overfat girls satisfied with own body shape was only 0.5% (n = 3) this group was not considered in this analysis.

## Discussion

The main findings of this study were: (1) many overfat boys were satisfied with their body image while practically all overfat girls reported to wish a thinner body; and (2) meal patterns and food consumption were associated with body dissatisfaction among overfat adolescents. In both genders, overfat adolescents that wished a thinner body were more likely to declare ≤3 eating occasions per day than normal-fat adolescents, and also than overfat boys satisfied with their own body image. Overfat girls that wish to be thinner skipped breakfast more frequently than normal-fat girls. Overfat boys and girls that wished a thinner body reported lower consumption of several food groups than normal-fat adolescents and overfat boys satisfied with their own body image (i.e. breakfast cereals, pasta and rice dishes, other oils and fats, high fat foods, soft drinks and chocolates in boys; and dairy products and chocolates in girls). Consequently, the overfat adolescents studied misreported their energy intake more often than normal-fat peers, and overfat adolescents dissatisfied with their body shape showed lower energy intake than normal-fat and satisfied peers. Restrictive eating practices related to a preoccupation with a slim image have been also reported among adolescents [38-40].

### Gender differences in body satisfaction

Boys and girls perceive their bodies in a different way [41]. It has been extensively previously reported that the current ideal male body is lean but highly muscular, characterised by a “well-developed chest and arms, with wide shoulders tapering down to a narrow waist” [42]. Thus, whereas boys with lower BMI and BF preferred a stronger muscular body, girls showed a preference for a slim body shape [41,43]. However, boys with elevated adiposity also showed a preference for a slim body shape; in fact, they have been most likely to have negative feelings about their bodies [44]. It has been suggested that adolescents who are heavy and perceive themselves as overfat may have actively tried to lose weight [45], whereas adolescents who do not perceive themselves as heavy raise concerns may be less motivated to take steps to lose weight [46]. Moreover, despite that adolescents denigrate overweight and obesity, a decrease in body dissatisfaction has been suggested in studies among young people [47].

It has been also recognized that to be worried about body image is especially acute in puberty [48], but also that body image is an important target of intervention to improve subjective health in adolescence [49]. It has been pointed out that some level of body dissatisfaction may be beneficial for individuals with average or above-average weight, as it may lead to healthy weight management behaviours such as increased intake of fruits and vegetables and regular physical activity [50,51]. To understand how body shape satisfaction affects meal patterns, food preferences and the overall adolescent diet is a key issue for the development of strategies aimed at influencing dietary behaviour. Accordingly, findings from the present study will be useful to understand relationships between body image and eating patterns.

### Meal patterns and food consumption are associated with body image

A previous study reported that normal-fat adolescents were more likely to follow a Western dietary pattern than a Mediterranean dietary pattern, and the wish to have a thinner body shape was associated with a low consumption of the Western dietary pattern [52]. Moreover, it has been also reported that parallel to the omission of meals, it may be possible that overfat adolescents that wished to be thinner avoided the consumption of several foods to counteract being overfat; boys and girls have been reported to avoid sweets and salty snack consumption to counteract being overweight [3]. Accordingly, a study conducted among 3055 Massachusetts high school students (aged 16 ± 1.2 years) found that adolescents attempted to lose weight consumed fewer servings of fatty foods, but they did not increase fruit and vegetable consumption; to lose weight, they also ate few servings of desserts, whereas to gain weight they ate more servings of these foods [16]. Previous findings showed that girls tried to lose weight eating few servings of meat, fries, chips, and dessert foods, whereas to gain weight they consumed few servings of fruit and green salad and increased the consumption of fries and chips [16]. A study conducted among 1220 Costa Rican adolescents (aged 12–18 years) showed that body image was associated with a high consumption of high-calcium and saturated fat foods, iron rich foods, and fruits and vegetables [53]. Our results suggest that adolescents that wish a thinner body decreased consumption of typical-Western-diet foods, but they did not increase consumption of fruits and vegetables, which may reflect a diet restriction rather than eating healthier food as a method to lose weight.

Overall, the current results highlight the importance of body image on adolescent nutritional habits and food choices. Particularly, a restriction of typical-Western-diet foods is associated with a wish to be thinner among overfat adolescents. A task for future research could be to include assessments of body image to better understand the prospective and concurrent contributions of body image to food consumption patterns among normal-weight, overweight and obesity adolescents. Our data could be useful to practitioners in targeting educational messages for individuals’ specific eating patterns and to community planners in encouraging the availability of a healthy dietary pattern.

## Conclusions

Many overfat boys were satisfied with their body image while practically all overfat girls reported wishing a thinner body. Meal patterns and food consumption were associated with body dissatisfaction and overfat status among adolescents.

### Strengths and limitations

This study has some limitations. The difficulties for assessing food intake among young people are well known but it should not serve as a deterrent to pursue this line of research. Moreover, BF was calculated using Slaughter et al. equations [19], which have been previously reported [20]. This study did not take into account pubertal development; however, a previous study [51] classified adolescents according to their pubertal stage and divided boys in two groups: pubertal (12–14 y.o.) and post-pubertal (15–17 y.o.). Moreover, it should be noted that we cannot ignore that adolescents that wish to be thinner could overestimate healthy foods consumption and underestimate unhealthy foods consumption; it has been well documented that people with high relative weight usually underreported their food intake [11]. Finally, we cannot infer causality because of the cross-sectional design of the study.

This study also has several strengths. New data is provided about the association between body image and food consumption patterns among adolescents according to their body composition. Specifically, it provides data evaluating the association between food consumption and dissatisfaction with overfat status among adolescents, which is scarce in this age group. Moreover, most of the previous studies in adolescents analyzed differences in perception of body image and weight concerns according to gender [49], ethnic and social differences [41], and overweight and obesity status, showing that BMI is positively related to body dissatisfaction [43-45]. However, in the present study, the association between body image and food consumption patterns according to body composition has been demonstrated. Thirdly, the use of BMI for age to define being overweight and obesity in children and adolescents is well established for both clinical and public health applications [54,55]. However, it has been recognized that elevation of BMI does not always equate to increased adiposity because it does not distinguish between BF mass and lean body mass [56], whereas the FMI has a high accuracy level for overweight screening [23]. Accordingly, after several statistically known potential confounding factors were controlled in this study, the adolescent population was classified according to both BMI and FMI, as it has been published elsewhere [57].

## Competing interests

The authors declare that they have no competing interests.

## Authors’ contributions

MMB, JP and JAT conceived, designed, devised and supervised the study, MMB, JP and JAT collected and supervised the samples. MMB and JAT analysed the data and wrote the manuscript. AP and JAT obtained funding. All authors read and approved the final manuscript.

## Pre-publication history

The pre-publication history for this paper can be accessed here:

http://www.biomedcentral.com/1471-2458/13/1104/prepub
